# Triphasic Oxygen Storage in Wet Nanoparticulate Polymer of Intrinsic Microporosity (PIM-1) on Platinum: An Electrochemical Investigation

**DOI:** 10.1021/acsami.4c04459

**Published:** 2024-07-12

**Authors:** Maisa Azevedo Beluomini, Nelson Ramos Stradiotto, Maria Valnice Boldrin Zanoni, Mariolino Carta, Neil B. McKeown, Philip J. Fletcher, Sunanda Sain, Zhongkai Li, Frank Marken

**Affiliations:** † Department of Chemistry, University of Bath, Claverton Down, Bath BA2 7AY, U.K.; ‡ Institute of Chemistry, São Paulo State University (UNESP), 14800-060 Araraquara, São Paulo, Brazil; § Department of Chemistry, Faculty of Science and Engineering, Swansea University, Singleton Park, Swansea SA2 8PP, U.K.; ∥ EaStCHEM School of Chemistry, University of Edinburgh, Joseph Black Building, David Brewster Road, Edinburgh, Scotland EH9 3JF, U.K.; ⊥ Materials & Chemical Characterisation Facility, MC^2^, University of Bath, Claverton Down, Bath BA2 7AY, U.K.

**Keywords:** triphasic gas storage, diffusion layer, energy storage, electrocatalysis, oxygen evolution

## Abstract

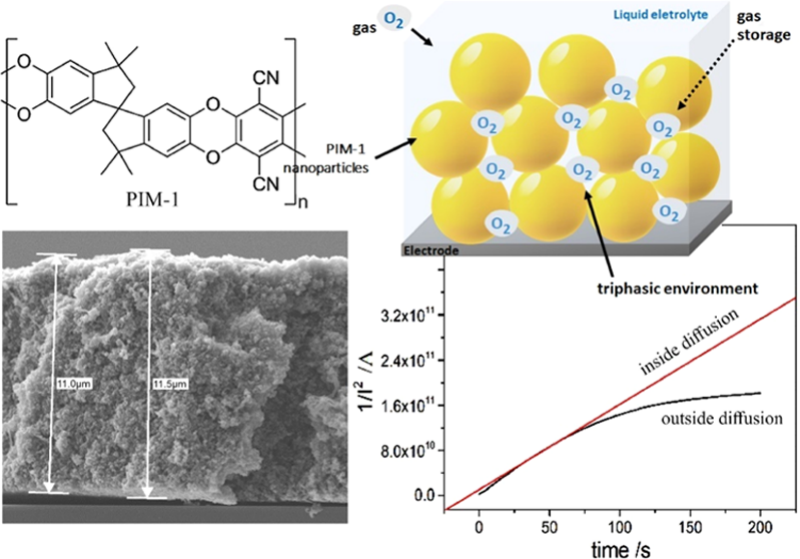

The triphasic interaction of gases with electrode surfaces immersed in aqueous electrolyte is crucial in electrochemical technologies (fuel cells, batteries, sensors). Some microporous materials modify this interaction locally via triphasic storage capacity for gases in aqueous environments linked to changes in apparent oxygen concentration and diffusivity (as well as activity and reactivity). Here, a nanoparticulate polymer of intrinsic microporosity (PIM-1) in aqueous electrolyte is shown to store oxygen gas and thereby enhance electrochemical signals for oxygen reduction in aqueous media. Oxygen reduction current transient data at platinum disk electrodes suggest that the reactivity of ambient oxygen in aqueous electrolyte (typically *D*_oxygen_ = 2.8 × 10^–9^ m^2^ s^–1^; *c*_oxygen_ = 0.3 mM) is substantially modified (to approximately *D*_app,oxygen_ = 1.6 (±0.3) × 10^–12^ m^2^ s^–1^; *c*_app,oxygen_ = 50 (±5) mM) with important implications for triphasic electrode processes. The considerable apparent concentration of oxygen even for ambient oxygen levels is important. Potential applications in oxygen sensing, oxygen storage, oxygen catalysis, or applications associated with other types of gases are discussed.

## 1 Introduction

**Figure 1:**
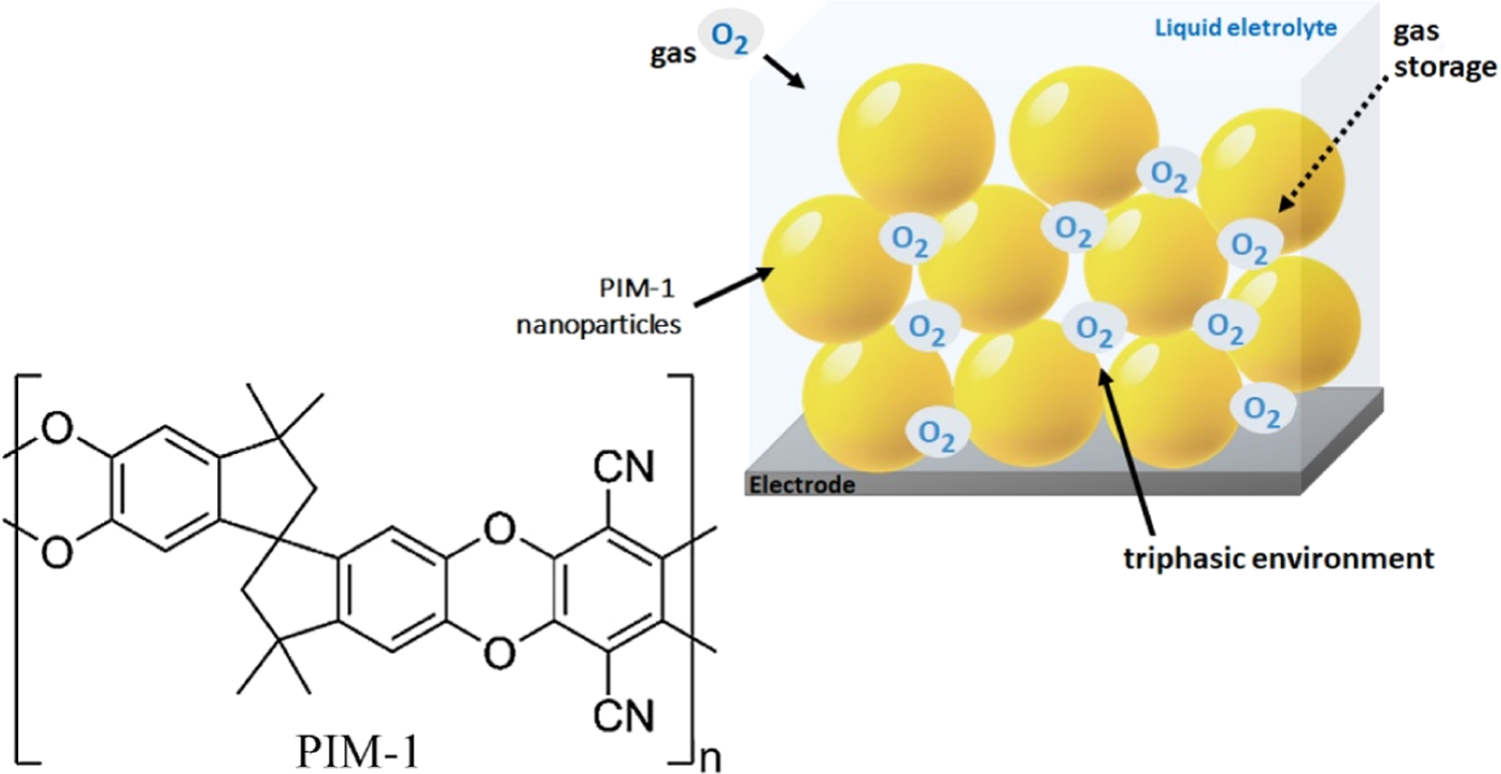
Molecular structure of PIM-1 and illustration of PIM-1 nanoparticles packed at the electrode | solution interface creating a triphasic gas | liquid | solid environment.

The oxygen reduction reaction has been carefully and systematically studied in the context of sensing,^1^ hydrogen peroxide production,^2^ in battery technologies,^3^ in fuel cells,^4^ or in organic electrosynthesis.^5^ One particular challenge for this technically important reduction reaction is linked to the relatively low solubility of oxygen in aqueous media typically ranging from 0.2 to 0.3 mM in the presence of ambient oxygen.^6^ In order to enhance the reactivity/solubility of oxygen (or other gases) in aqueous media (at ambient temperature), either pressure can be applied or solubility enhancing additives can be applied. In a recent report Erdosy et al.^7^ highlighted the ability of some microporous materials (MOFs, zeolites) to considerably enhance the apparent oxygen solubility in aqueous media. Similarly, we have recently shown that polymers of intrinsic microscopy (PIMs) and in particular PIM-1 (see molecular structure in Figure 1) provide a material for localized triphasic gas storage (directly at the electrode surface) in aqueous media for example for hydrogen^8^ or for oxygen.^9^ This localized gas storage at the electrode surface changes local oxygen activity, and therefore it potentially changes reaction rates or even reaction pathways at the electrode.

Polymers of intrinsic microporosity (PIMs) are molecularly rigid and contorted polymers with high specific surface area and selective permeability.^10−12^ Due to these properties, PIMs have been extensively studied for applications in separation membranes, catalysis, and in gas storage.^13^ PIMs provide microporous environments for binding molecular guests without electrical conductivity and without additional redox functionality.^14−16^ PIMs have found new applications in wet (electrochemical) conditions either in aqueous media for sensing,^17^ in gas diffusion electrodes,^18^ or when immersed in organic solvents for example in batteries.^19,20^ Recently, studies have shown that PIM-1 nanoparticles are effective as components for multiphase electrode surfaces.^21^ They can be utilized to store oxygen gas and enhance electrochemical signals for oxygen reduction.^22^ These PIM-1 nanoparticles immersed in water can act as a reservoir for oxygen, providing additional oxygen for the electrochemical reduction. However, the key storage parameters, the apparent concentration of oxygen (*c*_app,oxygen_) and the apparent diffusion coefficient (*D*_app,oxygen_) still need to be evaluated. In particular the *c*_app,oxygen_ parameter is important as a “storage” parameter describing gas behavior in aqueous media. By storing the gas evolved at an electrode, bubble formation can be avoided.^23^

In this report, chronoamperometry transient data for oxygen reduction at platinum electrodes are measured to give both apparent concentration and apparent diffusivity data for oxygen at the electrode surface. Data are obtained as a function of PIM-1 nanoparticle film thickness at the platinum electrode surface. The current transients are shown to switch as a function of time between internal diffusion (within the wet PIM-1 nanoparticle film) and external diffusion in the aqueous electrolyte. Combining data for different thicknesses of PIM-1 deposits allows both *c*_app,oxygen_ and *D*_app,oxygen_ within the PIM-1 nanoparticle films to be determined at the limit of high film thicknesses. It is demonstrated that PIM-1 can increase the local concentration of oxygen by 2 orders of magnitude without an external increase in pressure. These results demonstrate that “wet gas storage” is possible directly at electrode surfaces and that traditional limits of solubility in aqueous are readily overcome in the design of new types of triphasic electrode processes. These results imply that very similar storage effects are possible for other types of poorly water-soluble gases.

## 2 Experimental Section

### 2.1 Reagents

Monobasic sodium phosphate (≥98.0%), dibasic sodium phosphate (≥99.0%), methanol and chloroform were obtained from Sigma-Aldrich and used without further purification. PIM-1 (2,3,5,6-tetrafluorophthalonitrile-3,3,3′,3′-tetramethyl-1,1′-spirobisindane-5,5′,6,6′-tetrol copolymer, Sigma-Aldrich 918768, monomer molecular weight 460 g mol^–1^, molecular weight typically 70 KD) was synthesized using a method described in the literature.^24^ Argon and oxygen were purchased from BOC UK (Pureshield). Deionized and filtered water (18.2 MΩ cm at 20 °C) obtained from a Thermo Scientific water purification system, was used to prepare solutions. All experiments were conducted at a room temperature of 20 ± 2 °C. A 0.1 mol L^–1^ concentration of phosphate buffer solution pH 7 was used as a background electrolyte solution for all experiments.

### 2.2 Instrumentation

A potentiostat system (Autolab GPSTAT12, EcoChemie, The Netherlands) was employed with a Pt wire counter electrode and a KCl-saturated calomel reference. The working electrode was a platinum disk electrode (2 mm diameter). A conventional three-electrode electrochemical cell was employed. The electrode modified with PIM-1 nanoparticles was characterized using a field emission scanning electron microscope (FE-SEM, Jeol JSM-7900F) with an accelerating voltage of 5.0 kV. Particle size analysis was performed with ImageJ software. Surface area was determined by BET analysis with N_2_ isotherms obtained at 77 K using an Autosorb iQ-C-MP-AG (Quantachrome, Anton Paar gas sorption analyzer). Samples were degassed *in situ* at 353 K for 12 h before the measurement. Pore size distributions were estimated using nonlocal density functional theory (NLDFT) using a slit-pore model for N_2_ (BOC Ltd.) at 77 K.

### 2.3 Procedures

PIM-1 nanoparticles were synthesized with typically 35 nm diameter using an antisolvent precipitation method, as reported previously.^9^ Briefly, the PIM-1 polymer was dissolved in 2 mL of chloroform at a concentration of 1 mg mL^–1^. The solution was added dropwise into 20 mL of methanol with vigorous stirring for another 12 h. The PIM-1 solution was centrifuged for 30 min at 5000 rpm followed by the removal of excess methanol. The PIM-1 nanoparticles were subsequently redispersed in methanol using an ultrasonication process. To prepare nanoparticulate films, a volume of 5 μL (4 mg mL^–1^) of PIM-1 solution in methanol, equivalent to 20 μg PIM-1 (other quantities of PIM-1 used in this study were calculated proportionally), was drop-coated onto the platinum electrode to dry at room temperature.

### 2.4 Characterization

**Figure 2:**
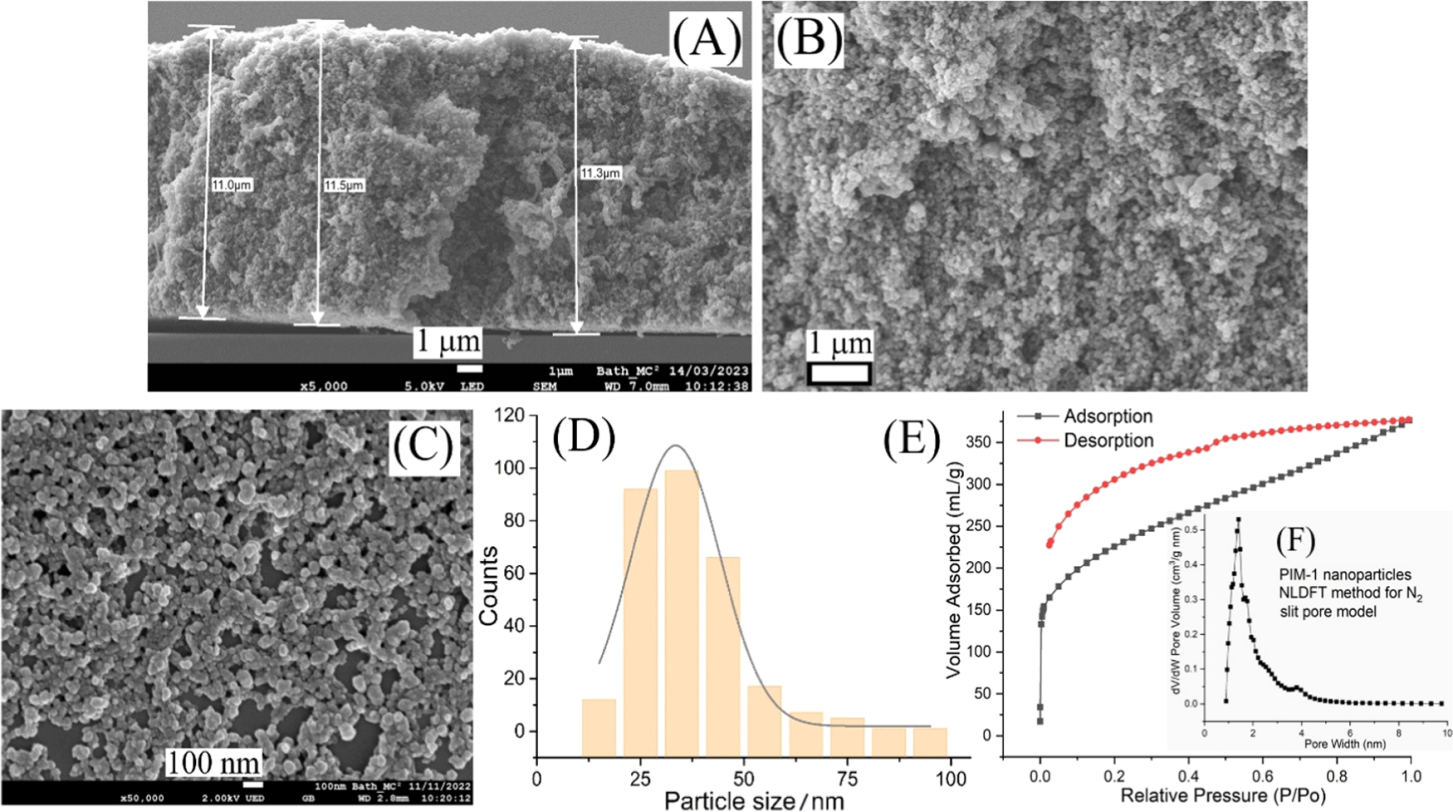
(A) Scanning electron microscopy (SEM) of cross-sectional view of the silicon wafer coated with PIM-1 nanoparticles (600 μm on approximately 7 × 10^–6^ m^2^). (B) As above with higher magnification. (C) SEM of a thin nanoparticle layer on a silicon substrate with (D) histogram of particle size data showing typically 35 nm diameter particles. (E) Nitrogen adsorption (BET) isotherm (surface area is 764.4 m^2^g^–1^) and (F) micropore size distribution determined by NLDFT (slit pore model for N_2_ at 77 K).

Figure 2A shows a typical scanning electron microscopy (cross-sectional SEM) image of for a deposit of 600 μg of PIM-1 nanoparticles on an approximately 7 × 10^–6^ m^2^ area (employing a silicon wafer substrate to replicate the inlaid Pt disc electrode surface). A porous film of nanoparticles is observed covering the surface with a thickness of approximately 12 μm. Figure 2B shows the nanoparticle film in higher magnification. Figure 2C shows an SEM image of a thinner layer of nanoparticles distributed on a surface. Image analysis allows the typical nanoparticle diameter to be estimated (Figure 2D) as 35 nm. Figure 2E,F show nitrogen adsorption (BET) surface analysis data for the PIM-1 nanoparticles consistent with previous reports for bulk and electrospun PIM-1^25,26^ and consistent with a surface area of 764.4 m^2^g^–1^.

The value of the approximate film thickness estimated from SEM data, was employed to calibrate the variable thickness of films deposited onto the platinum disk electrode. Table 1 summarizes the thicknesses of the nanoparticulate film for different amounts of PIM-1 deposited onto the electrode surface.

**Table 1:** Thickness Variation of PIM-1 Nanoparticles on Pt Electrode (Estimated Based on Calibration with Cross-Sectional Electron Microscopy Images; Figure 2A)

PIM-1 weight of deposit/μg	**approximate thickness** δ/μm*
20	0.4 (±0.08)
50	1 (±0.2)
100	2 (±0.4)
200	4 (±0.8)
400	8 (±1.6)
600	12 (±2.4)

* Errors in terms of roughness and repeatability estimated as ±20%.

## 3 Results and Discussion

### 3.1 Detection of Oxygen Stored in Nanoparticulate PIM-1: Cyclic Voltammetry

**Figure 3:**
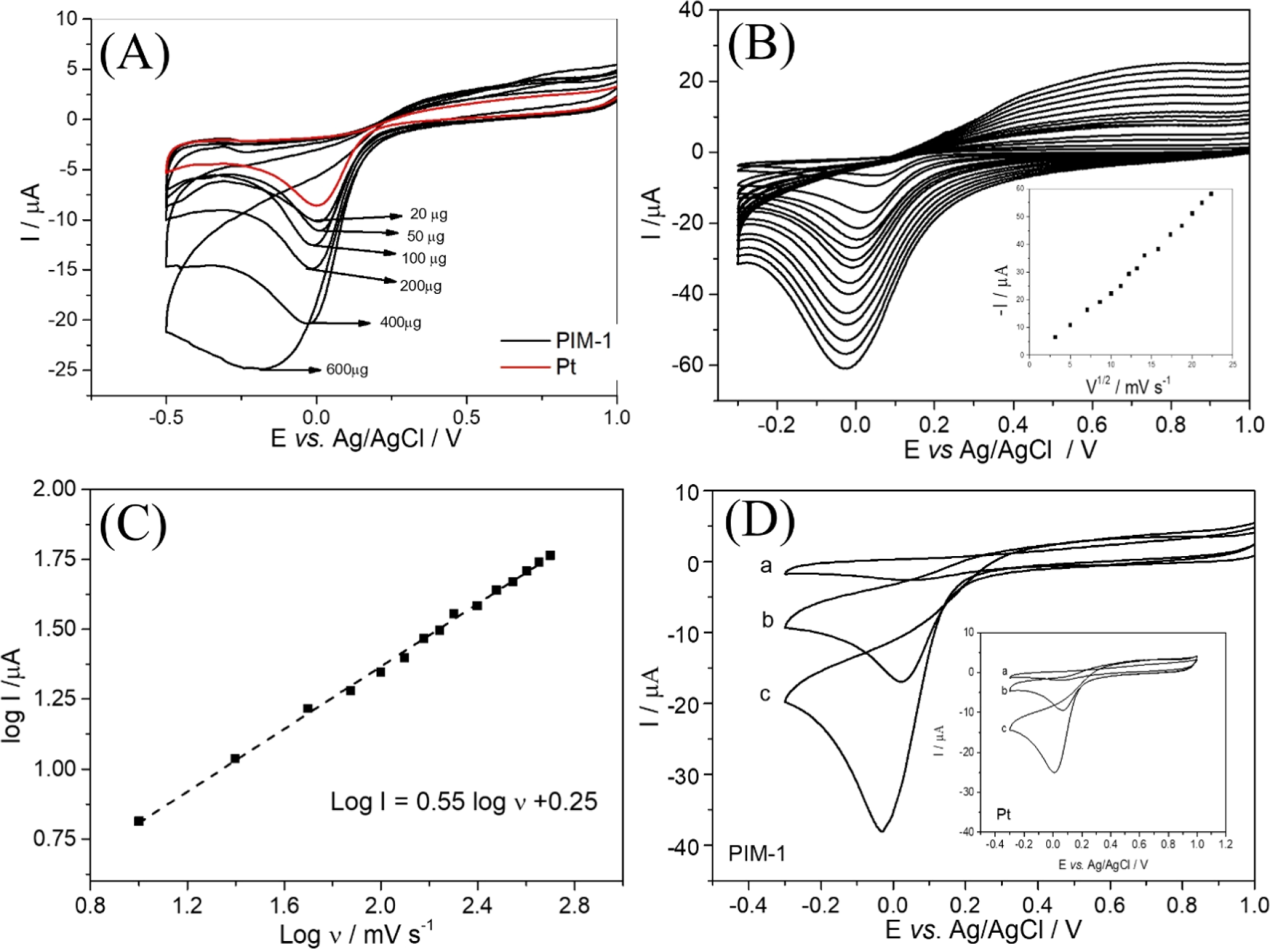
(A) Cyclic voltammograms (scan rate 50 mV s^–1^) for a 2 mm diameter Pt electrode immersed in 0.1 mol L^–1^ phosphate buffer pH 7. Data are shown for the bare electrode (red) and for different amounts of PIM-1 nanoparticle deposits (20–600 μg). (B) Cyclic voltammograms (scan rates 10–500 mV s^–1^) for 200 μg PIM-1 nanoparticles on a 2 mm diameter Pt disk. Insert: plot of peak currents of oxygen reduction versus square root of scan rate. (C) Double-logarithmic plot of the peak current for oxygen reduction versus the scan rate. (D) Cyclic voltammograms (scan rate 50 mV s^–1^) for 200 μg PIM-1 nanoparticles on a 2 mm diameter Pt disk electrode in argon atmosphere (a), ambient air (b), and in pure oxygen atmosphere (c). Insert: the same conditions but for a bare Pt electrode.

In order to evaluate the ability of PIM-1 nanoparticles to store oxygen gas, different amounts of PIM-1 nanoparticles (different thicknesses) were deposited onto a 2 mm diameter Pt disk electrode. A solution containing 0.1 mol L^–1^ phosphate buffer pH 7 usually with ambient oxygen was employed in the voltammetric measurements. Cyclic voltammograms (50 mV s^–1^ scan rate) in Figure 3A show peaks for the oxygen reduction reaction at 0.0 V vs Ag/AgCl. For the bare Pt electrode (red curve) a peak current of −8 μA is observed. In the presence of varying amounts of PIM-1 nanoparticles on the Pt disk electrode the reduction current is significantly enhanced. With 600 μg PIM-1 nanoparticles the current triples and the peak broadens. This increase in current (and charge) can be assigned either to faster diffusion or (perhaps more likely) an increased concentration of oxygen accumulated locally at the electrode surface (or to both) in the presence of the PIM-1 nanoparticles.

#### 3.1.1 Effect of the Scan Rate on Current Peaks

The influence of scan rate (υ) on the oxygen reduction peak currents in the presence of PIM-1 nanoparticles on Pt was investigated. The scan rate was varied from 10 to 500 mV s^–1^ (Figure 3B). The current peak at 0.0 V vs Ag/AgCl corresponds to oxygen reduction in phosphate buffer solution pH 7 (eq 1).
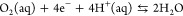
1

The peak currents increase with scan rate, and the peak potentials shift toward more negative potentials. There is a linear relationship between the peak current versus square root of the scan rate (Figure 3C,D) indicative of a diffusion-controlled process. The equation for peak current versus scan rate can be expressed as log*I*(μA) = 0.55 log υ (mV s^–1^) + 0.25 (*R* = 0.998) consistent overall with a diffusion-controlled process.^27^ This diffusion process could be entirely within the PIM-1 nanoparticle film for sufficiently small diffusion coefficients (*vide infra*).

#### 3.1.2 Effect of Gas Composition

Upon modifying the gas environment during the experiments (by 20 min gas purging with either oxygen or argon gas), significant (and anticipated) effects on the oxygen reduction reaction were observed. Figure 3D shows the data obtained from testing the PIM-1 nanoparticle coated Pt disk electrode. The oxygen reduction peak current is consistent with the oxygen content in the gas phase. The inset shows data for a bare Pt electrode for argon, ambient air, and for pure oxygen, revealing very similar changes in the reduction peak current linked to variations in oxygen concentration. Therefore, oxygen gas concentration equilibration occurs from the gas phase to the liquid phase and finally into the solid PIM-1 phase. The question arises whether the oxygen reduction peak current in the presence of PIM-1 nanoparticles is affected by changes in apparent diffusion coefficient or by changes in apparent concentration (or both). Chronoamperometry experiments can be employed to answer this question.

### 3.2 Detection of Oxygen Stored in Nanoparticulate PIM-1: Chronoamperometry

#### 3.2.1 Effect of the Amount of PIM-1 Deposit

**Figure 4:**
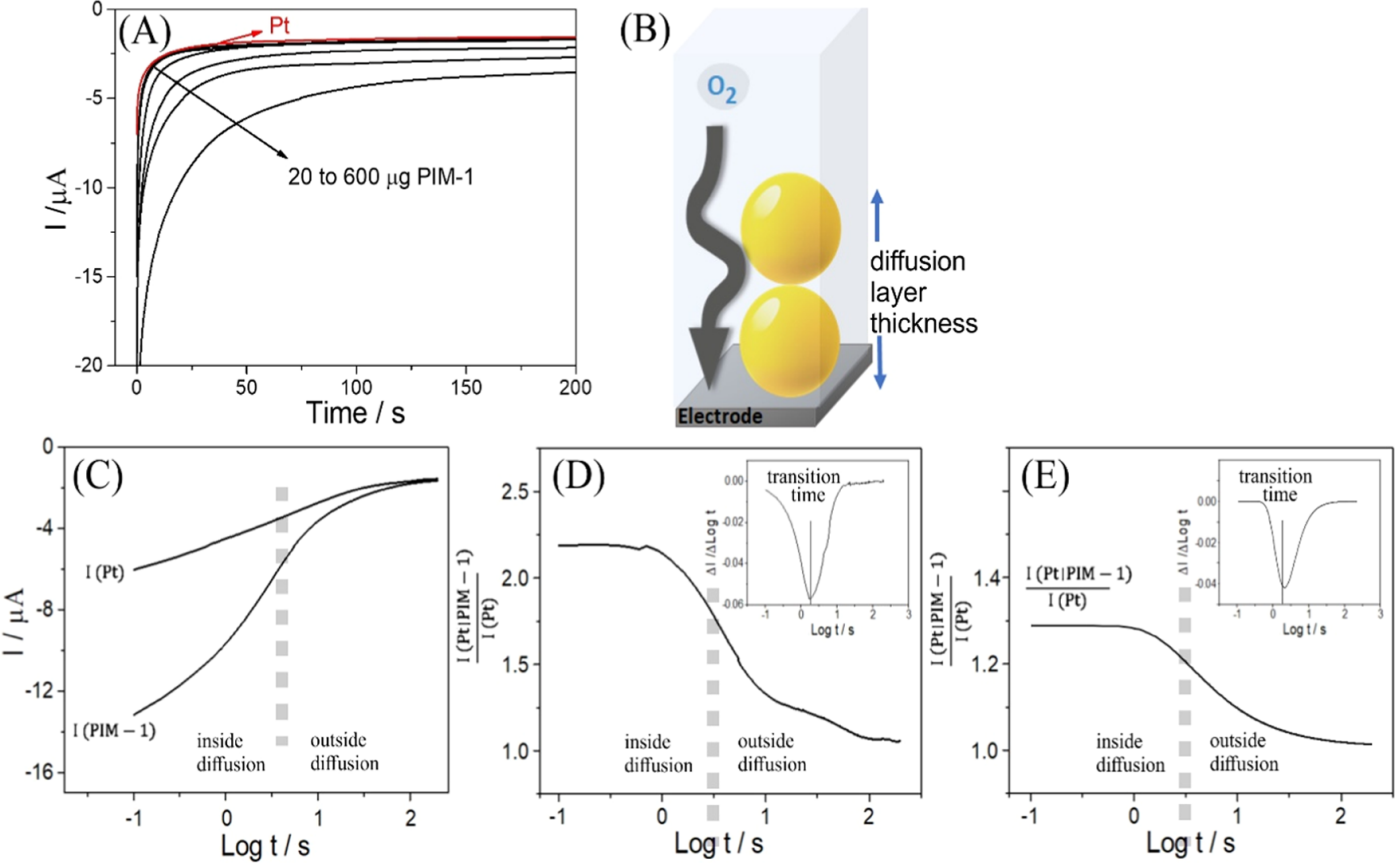
(A) Chronoamperometric response for a 2 mm diameter Pt disk electrode bare or coated with PIM-1 nanoparticles (20, 50, 100, 200, 400, and 600 μg PIM-1) immersed in 0.1 mol L^–1^ phosphate buffer pH 7 with applied potential −0.2 V vs Ag/AgCl. (B) Illustration of oxygen diffusion through a layer of PIM-1 nanoparticles. (C) Oxygen reduction current transients versus logarithm of time for chronoamperometry at a bare Pt electrode and a PIM-1 nanoparticle covered Pt disk electrode (50 μg PIM-1). (D) Plot of the current ratio *I*_PIM-1|Pt_/*I*_Pt_ vs Log *t*. Insert: first derivative to demonstrate the peak to identify the transition from inside diffusion to outside diffusion. (E) As before, but for theoretical model data (see text) for *D*_oxygen_ = 2.8 × 10^–9^ m^2^ s^–1^, *D*_app,oxygen_ = 2.5 × 10^–13^ m^2^ s^–1^, *c*_oxygen_ = 2.8 mM, *c*_app,oxygen_ = 28 mM, and a film thickness of δ = 1 μm.

Chronoamperometry data were recorded for approximately 0, 20, 50, 100, 200, 400, and 600 μg PIM-1 nanoparticles (see Table 1) deposited onto a 2 mm diameter Pt disk electrode immersed in 0.1 mol L^–1^ phosphate buffer (pH 7) with an applied potential of −0.2 V vs Ag/AgCl within the oxygen reduction potential region (Figure 3). Figure 4A shows chronoamperometry transient data over 200 s for the reduction of ambient oxygen. The presence of the PIM-1 nanoparticles clearly substantially increases the reduction current and even after 200 s the higher current remains significant indicative of more oxygen reaching the electrode surface. Replotting the data with a logarithmic time axis (Figure 4C) shows that a transition occurs where the PIM-1 nanoparticle coated electrodes transition from diffusion inside the PIM-1 film to diffusion outside of the PIM-1 nanoparticle film. The ratio of transient currents is plotted in Figure 4D clearly showing the transition from inside to outside diffusion.

By plotting the current data versus logarithm of time, the diffusional transport first within the PIM-1 nanoparticle region and second within the external electrolyte phase can be seen more clearly (Figure 4C). When plotting the ratio of current in the presence and in the absence of PIM-1 (as a dimensionless parameter), sigmoidally shaped plots are observed (Figure 4D) that step from a value of approximately 2.2 (for short time) to 1.0 (for longer time).^28^ The transition from diffusion inside to outside is not smooth due to porosity and heterogeneity in the polymer film, but a first derivative of the dimensionless parameter plot shows a clear peak indicating the point in time where the transition occurs. It is possible to evaluate the transition time for each PIM-1 film thickness and then evaluate the apparent diffusion coefficient based on one-dimensional Fickian diffusion (eq 2).
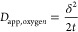
2

In order to rationalize/verify this methodology, the mathematical model by Peerce and Bard^29^ is employed (eq 3 showing the Cottrell equation for planar bulk diffusion; eq 4 for the case of diffusion through a thin film into the bulk; with *n*, the number of electrons transferred per molecule diffusing to the electrode, *F*, the Faraday constant, *A*, the electrode area, *c*, the bulk concentration, *D*, the diffusion coefficient in solution, and *t*, the time; introducing a film with thickness δ on the electrode surface, and parameter  with *K* = *c*_app_/*c*, the partitioning constant for the reactant) to produce theoretical model data (in Figure 4E) for *D*_oxygen_ = 2.8 × 10^–9^ m^2^ s^–1^, *D*_app,oxygen_ = 2.5 × 10^–13^ m^2^ s^–1^, *c*_oxygen_ = 2.8 mM, *c*_app,oxygen_ = 28 mM, and a film thickness of δ = 1 μm (*vide infra*). The transition from inside to outside diffusion for experimental data and for theory data appear at a very similar time, which justifies the use of the approximate eq 2. However, the physical meaning of *D*_app_ in the Peerce-Bard model is different to that in the experiment due to roughness and the heterogeneity in the PIM-1 nanoparticle film. Therefore, different film thicknesses lead here to systematic changes in *D*_app_ converging to a limit for thicker films (*vide infra*).
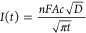
3
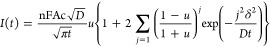
4

Table 2 summarizes thickness, transition time, and *D*_app,oxygen_ data. The diffusion coefficient for oxygen is decreased dramatically when compared to the diffusion coefficient in aqueous electrolyte. There is a 3 orders of magnitude change/decrease and therefore diffusion of molecular oxygen in the PIM-1 nanoparticle film is extremely slow, comparable with diffusion in other types of polymers. For example, a diffusion coefficient of below 10^–12^ m^2^ s^–1^ can be compared with that observed in solid poly ethylene-terephthalate (PET).^30^

**Table 2:** Experimental Data from Chronoamperometry for Oxygen Reduction at Pt Disk Electrode with PIM-1 Nanoparticle Deposits Immersed in Ambient 0.1 M Phosphate Buffer pH 7^a^

**amount of** PIM-1/μg	**approx transition** time/s	**film thickness** δ/μm^d^	***D***_**app**,**oxygen**_**/m**^**2**^ **s**^**–1**^^d^	**approx**. **cottrell** slope/A^**–2**^**s**^**–1**^	**apparent oxygen concentration*****c***_**app**,**oxygen**_**/mM**^d^
0	-c	0.0	2.8 × 10^–9^	8.0 × 10^9^	0.3^b^
20	0.5	0.4 (±0.08)	1.9 (±0.1) × 10^–13^	1.5 × 10^10^	27 (±3)
50	2	1 (±0.2)	2.5 (±0.1) × 10^–13^	6.6 × 10^9^	28 (±3)
100	4	2 (±0.4)	5.0 (±0.1) × 10^–13^	7.4 × 10^9^	34 (±3)
200	10	4 (±0.8)	8.0 (±0.2) × 10^–13^	1.6 × 10^9^	41 (±4)
400	21	8 (±1.6)	1.6 (±0.3) × 10^–12^	7.4 × 10^8^	43 (±4)
600	45	12 (±2.4)	1.6 (±0.3) × 10^–12^	5.1 × 10^8^	50 (±5)

a Error in the data is assumed to be dominated by error in thickness *d* (estimated as ±20%) propagating to error in *D*_app_ (±20%) and then to *c*_app_ (±10%).

b For the Pt electrode without PIM-1 addition an apparent oxygen concentration of 0.3 mM is obtained (considering *D*_oxygen_ = 2.8 × 10^–9^ m^2^ s^–1^;^9^ estimated error ±0.1 mM).

c The observed transition is likely to be associated with convection effects are the bare electrode.

d Errors in thickness in terms of roughness and repeatability estimated as ±20% which propagates to error in *D*_app_ as ±20% and in *c*_app_ as ±10%.

**Figure 5:**
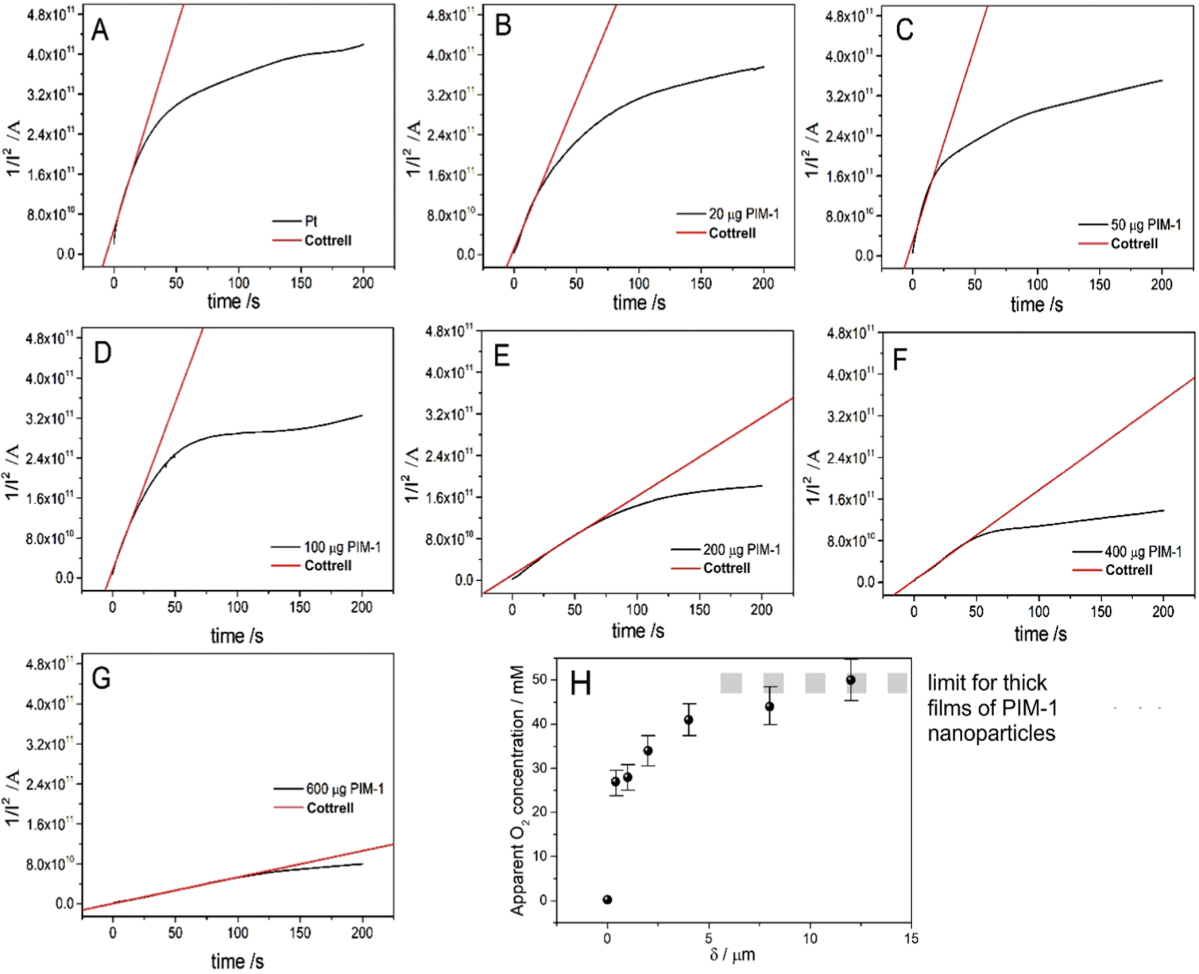
Cottrell plots (1/I^2^ versus time) for of different amounts of PIM-1 nanoparticles on a 2 mm diameter Pt disk electrode (for (A) 0, (B) 20, (C) 50, (D) 100, (E) 200, (F) 400, and (G) 600 μg PIM-1) immersed in a solution containing ambient 0.1 mol L^–1^ of phosphate buffer pH 7. Black line: experimental data; red line: fitting of Cottrell line. (H) Plot of the apparent oxygen concentration versus PIM-1 nanoparticle thickness limiting at approximately *c*_app,oxygen_ = 50 mM (errors estimated ±10%).

Next, by means of chronoamperometry, the currents flowing through the PIM-1 modified electrode during the early stages of the transient are investigated. Cottrell plots (1/I_2_ versus time) of the data (Figure 5) are fitted with the Cottrell line to match the early data points (red line). The Cottrell eq (eq 3) is employed to describe planar diffusion to the electrode surface. In this equation *F* is the Faraday constant (96485 C mol^–1^), *A* is the geometric area of the electrode (3.14 × 10^–6^ m^2^), *c* is the bulk concentration of the oxygen species, *n* is the number of electrons for each oxygen molecule diffusing to the surface (*n* = 4), and *D* is the diffusion coefficient in m^2^ s^–1^ (as evaluated based on the eq 2). The equation is transformed into eq 5 to show that the slope in the Cottrell plots in Figure 5 provide access to *c*^2^*D* and thereby (with *D* estimated) to the apparent concentration of oxygen.
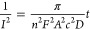
5

Table 2 summarizes the data. Figure 5H shows a plot of the apparent oxygen concentration in the PIM-1 film limiting at approximately 50 mM which is more than 2 orders of magnitude higher when compared to the concentration of oxygen in aqueous solution under ambient conditions. The observation of a limiting value for an increased thickness of PIM-1 nanoparticle film can be attributed to the apparent diffusion coefficient converging toward the true internal diffusion coefficient only for thicker films. The PIM-1 nanoparticles are able to store high amounts of gas (oxygen) locally at the electrode surface and this can affect the mechanism and activity of oxygen at the electrode surface. These observations are consistent for example with work by Erdosy^7^ on the enhanced oxygen solubility in aqueous solutions/dispersions.

#### 3.2.2 Effect Salt Concentration (PIM-1)

**Figure 6:**
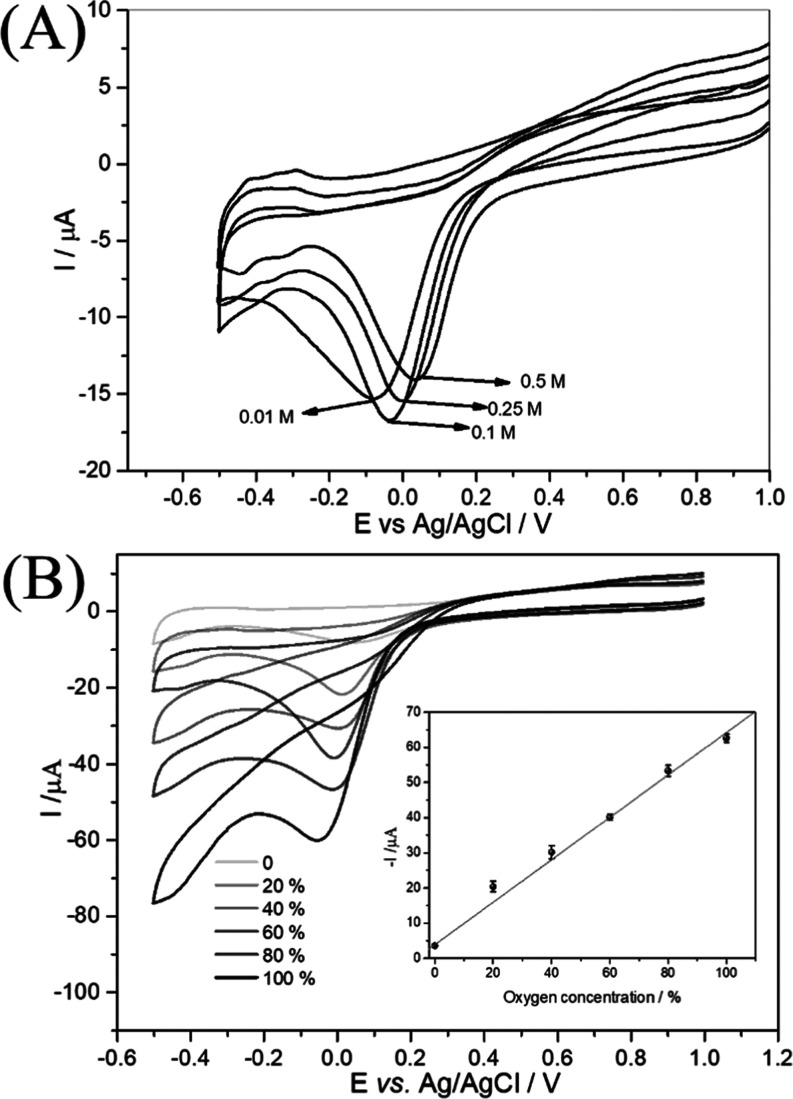
(A) Cyclic voltammograms (scan rate 50 mV s^–1^) for the reduction of ambient oxygen in 0.01, 0.10, 0.25, and 0.50 M phosphate buffer pH 7 at a PIM-1 nanoparticle coated Pt disk electrode (400 μg PIM-1). (B) Cyclic voltammograms (scan rate 50 mV s^–1^) of 400 μg PIM-1 deposited on a 2 mm diameter Pt disk electrode immersed in 0.1 mol L^–1^ of phosphate buffer pH 7 saturated with gas containing argon and oxygen. Insert: Calibration plot of the sensor for oxygen determination.

It is interesting to explore the effects of ionic strength (buffer concentration) on the triphasic gas storage capability of PIM-1 nanoparticles. Figure 6A shows cyclic voltammetry data for the reduction of ambient oxygen in 0.01, 0.10, 0.25, and 0.50 M phosphate buffer pH 7 at a PIM-1 nanoparticle coated Pt disk electrode (400 μg PIM-1). It is evident that the phosphate buffer concentration exerts some influence on the kinetically controlled reduction peak position, possibly due to a small shift in the potential for the reduction of the underlying platinum oxide to active platinum catalyst. However, the peak current and the apparent diffusivity/concentration of oxygen remained practically constant.

### 3.3 Detection of Oxygen Stored in Nanoparticulate PIM-1: Sensor Electrodes

In order to demonstrate experimental detection of oxygen, the dissolved oxygen concentration in the 0.1 mol L^–1^ of phosphate buffer pH 7 was controlled by mixing oxygen and argon gas prior to purging the solution. Different ratios of oxygen to argon (flowing into the solution to equilibrate for approximately 20 min) were controlled accurately using gas mass-flow controllers (MFC, Platon). The flow rate of oxygen gas was controlled to give 20, 40, 50, 80, and 100% partial pressure. The laboratory temperature was 21 ± 1 °C throughout all measurements. Figure 6B shows cyclic voltammograms for the reduction of oxygen at Pt disk electrode coated with 400 μg PIM-1. An oxygen reduction the peak was observed at −0.05 V vs Ag/AgCl. The current increased with oxygen concentration and the peak potential shifted slightly with increasing oxygen concentration. A linear calibration plot demonstrates the fact that the PIM-1 enhanced reduction current signals are directly proportional to the partial pressure of oxygen in the gas phase. This can be interpreted in terms of both oxygen and argon binding into PIM-1 in the same ratio as given by the purging gas. In other words, other types of gases will interact with PIM-1 nanoparticles in a similar way as oxygen. Electrode processes based, for example, on hydrogen oxidation, nitrogen reduction, or carbon dioxide reduction should be affected in a similar way.

In terms of practical applications, the observation of enhanced oxygen reduction currents and significantly enhanced local concentrations of oxygen at the electrode surface may lead to benefits in water-based electrochemical devices such as fuel cells, air-metal batteries, or oxygen sensors. Using PIM-1 nanoparticles to locally store oxygen can affect oxygen activity and reactivity at the electrode surface. Similar effects are anticipated for other types of gases and electrode processes.

## 4 Conclusions

The effects of PIM-1 nanoparticle deposits at a platinum electrode immersed in aqueous 0.10 M phosphate buffer pH 7 have been quantified. Oxygen gas reduction at the electrode is enhanced due to triphasic gas storage in PIM-1. In the presence of PIM-1 nanoparticles (i) the apparent solubility of oxygen in the aqueous phase increases from 0.3 to 50 (±5) mM (by more than 2 orders of magnitude) and (ii) the apparent diffusivity of oxygen decreases from approximately 10^–9^ to 10^–12^ m^2^ s^–1^ (by 3 orders of magnitude). Reactivity of oxygen gas at the electrode is considerably modified. Note that under 1 bar oxygen the apparent concentration of oxygen in the PIM-1 nanoparticle film reaches 0.25 M. In the future, better numerical simulation tools and better theoretical models (taking into account film heterogeneity) will be required for improved understanding of transport and storage processes. Furthermore, new experimental approaches need to be developed for quantitative gas adsorption measurement (by synchrotron or thermogravimetry techniques) in wet microporous media.

The effect of PIM materials on triphasic electrode processes has been reported previously for oxygen and for hydrogen gases. It is likely that the effect is general and potentially beneficial in other types of processes such as electrochemical nitrogen reduction or electrochemical carbon dioxide reduction with appropriate electrode | solution interface design. More generally, processes involving gas evolution such as hydrogen evolution and oxygen evolution are likely to be enhanced due to the “*in situ* storage” of gas close to the electrode surface. This phenomenon will allow gaseous products to be generated and stored similar to the case of battery energy storage (a “hydrogen battery”) but without the need for lithium and intercalation reactions based on transitional metal oxides. Further potential for application and for further structural evolution of PIM materials for enhanced performance and for specific applications is high.
